# Method for measuring the transpiration resistance of fruit and vegetables

**DOI:** 10.1016/j.mex.2024.103058

**Published:** 2024-11-16

**Authors:** Manfred Linke, Tuany Gabriela Hoffmann, Akshay D. Sonawane, Guido Rux, Pramod V. Mahajan

**Affiliations:** Leibniz Institute for Agricultural Engineering and Bioeconomy (ATB), Department Systems Process Engineering, Potsdam, Germany

**Keywords:** Fresh food, Horticultural, Transpiration, Respiration, Postharvest, Storage, Transpiration resistance determination for fruit and vegetables

## Abstract

This investigation explores the intricate relationship between postharvest quality losses in fruit and vegetables and the dynamic interplay of transpiration and respiration activities. It underscores the profound impact of inherent produce properties and postharvest environmental conditions on transpiration, inducing changes in both external appearance and internal quality, notably wilting. Despite their common use, produce-specific transpiration coefficients encounter limitations due to diverse assumptions in calculations. Surface conditions intricately link produce and air properties, necessitating a comprehensive understanding. Horticultural products, with high water content, undergo continuous water loss through transpiration, driven by the water potential difference between the product and ambient air. Transpiration encompasses tissue and boundary layer resistances, influenced by plant tissue properties and external factors. Fruits experiencing drought stress exhibit elevated tissue resistance, serving as a protective mechanism. Concurrently, boundary layer resistance, influenced by external parameters, significantly shapes postharvest behaviour. To address these complexities, a novel method developed allows separate analysis of produce properties, climate, and flow conditions. This innovative approach enhances the understanding of transpiration behaviour, providing a foundation for improved postharvest practices, technical configurations, and quality maintenance strategies.

• Direct method for tissue resistance and boundary layer resistance determination for fruit and vegetables.
• Non-destructive method to optimize postharvest by using produce as a sensor to ensure quality.

## Graphical abstract

**Figure:**
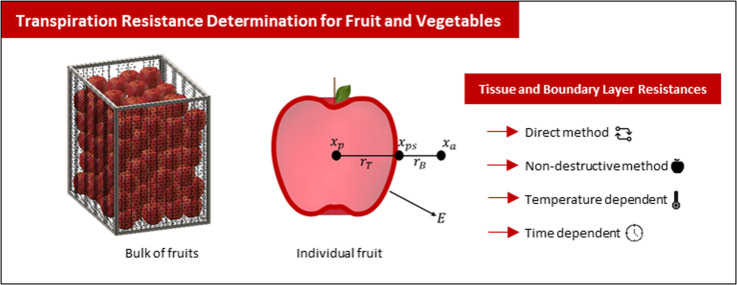

Specifications table

**Table:** 

Subject area:	Agricultural and Biological Sciences
More specific subject area:	Postharvest physiology in horticulture
Name of your method:	Transpiration resistance determination for fruit and vegetables
Name and reference of original method:	Not applicable.
Resource availability:	Not applicable.

## Background

Postharvest quality losses in fruit and vegetables are intricately linked to the transpiration and respiration activities influenced by both the inherent properties of the produce at harvest and the environmental conditions at the postharvest. Fruit and vegetables are subjected to varying climatic stresses during postharvest and undergo changes in both external appearance and internal quality due to the dynamic nature of transpiration, causing wilting. The transpiration rate is generally the amount of water that is discharged by transpiration into the atmosphere under certain conditions per unit of time. In the realm of postharvest physiology, a common practice is to describe transpiration behaviour using produce-specific transpiration coefficients, as documented by Sastry et al. [1] and Dickerson [2]. These coefficients, representing the reciprocal of resistance, are considered characteristic quantities. However, their utility is limited due to the wide array of assumptions made by different authors in their calculations. Each coefficient is valid within specific experimental conditions, characterized by the initial produce properties and the environmental parameters during experiments. Applying these coefficients to diverse postharvest scenarios assumes knowledge of air temperature, humidity, and flow conditions close to the produce.

Conditions on the fruit surface are intricately linked to produce properties, surrounding air properties (temperature, humidity), and flow conditions around the produce, as outlined by Linke and Geyer [3]. This intricate relationship underscores the need for a detailed understanding, as surface conditions may significantly deviate from the saturation state under certain circumstances. To characterize the transpiration properties of the fruit, the water vapour permeance is generally not used due to challenges in non-destructive measurement of epidermal layer thickness and the non-uniform water distribution inside the fruit. Horticultural products, with water content reaching up to 95 %, experience continuous water loss to the environment postharvest in a process known as transpiration. The driving force behind this process is the water vapour potential difference between the intercellular space of the product and the surrounding ambient air [4].

Transpiration is comprised of two resistances: tissue resistance and boundary layer resistance, collectively forming the total transpiration resistance, as proposed by Böttcher and Belker [5]. The tissue resistance is dictated by the nature of the plant tissue; with inner properties such as mesocarp and endocarp texture influencing this biological function. Fruits exposed to high drought stress, like those under sunlight, heat, dry air, and higher air velocities, exhibit significantly higher tissue resistance, acting as a protective mechanism against dehydration. On the other hand, fruits in low drought stress conditions, like tubers in moist soil, display lower tissue resistance. Boundary layer resistance, determined by external parameters such as shape, dimensions, surface texture, and environmental conditions like air velocity, temperature, and humidity, plays a crucial role in the postharvest behaviour of horticultural products. These climatic conditions directly influence the boundary layer resistance and, consequently, transpiration.

To address the intricate relationships between produce properties, climate, and flow conditions, a novel method developed at the Leibniz Institute for Agricultural Engineering and Bioeconomy (ATB) offers an innovative approach. This method allows the separate analysis of these factors, providing a more detailed understanding of transpiration behaviour and laying the foundation for improved postharvest practices, technical configurations, and quality maintenance strategies.

## Method details

The determination of the resistances in the water vapour pathway is based on modified Fick's law of diffusion in terms of resistances, describing the rate of water loss E as the ratio of a potential difference Δx to the total resistance $r_{tot}$ [6,7], as presented in Eq. (1). E is known as the area-related transpiration rate (in mg cm^−2^ h^−1^).

(1) $$E=\frac{x_{p}-x_{a}}{r_{T}+r_{B}}=\frac{\Delta x}{r_{tot}}$$

**Fig. 1:**
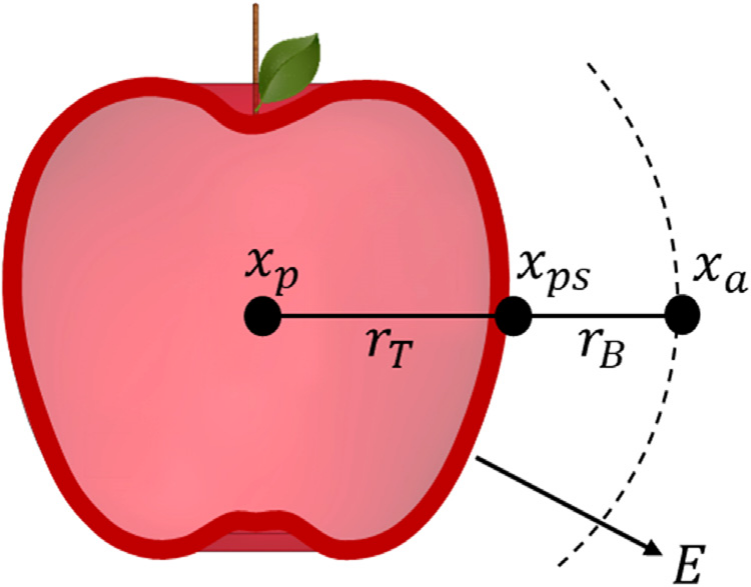
Schematic illustration of the components that act as the driving force for mass transfer.

The potential difference Δx as the driving force for mass transfer consists of two components: the water vapour concentration of the air in the centre of the produce (in intercellular spaces) $x_{p}$ and the water vapour concentration of the ambient air in sufficient distance to the produce $x_{a}$ (Fig. 1). It can be assumed that the air in the centre of fresh horticultural produce is saturated (100 % rH) or very close to the saturation state [4,8,9]. However, this argument is valid as long as there are no external indications of wilting (fresh fruit and vegetables).

The water vapour concentration of the air $x_{a}$ can be expressed as the partial pressure of water vapour, water potential or in terms of the absolute water content. For further considerations, the volume-related water content (in g of water per m^3^ of dry air) is used due to compatibility with the transpiration rate and the resistances.

The surface temperature of the fruit will be below the temperature of the surrounding air in particular at relatively low humidity, because during the evaporation process, heat will be removed from the site of evaporation [10]. The water vapour concentration of the ambient air $x_{a}$* is determined from the measured values of relative humidity, air temperature and barometric air pressure following the same basic relations (Table 1).

**Table 1:** Basic psychrometric relations for the determination of various air parameters from measured temperature, relative humidity and barometric pressure [11,17].

Equation	Formula	Unit	Description
(2^*)^	$p_{sa}=\;e^{\frac{\left(6.4142801\;+\;0.0996709\;\times \;T_{a}\right)}{\left(1\;+\;0.004197225\;\times \;T_{a}\right)}}$	Pa	Partial pressure of water vapour at saturation depending on temperature ($\;T_{a}$)
(3)	$p_{da}=\;p_{sa}\times \;(rH\;/100)$	Pa	Partial pressure of water vapour depending on saturation vapour pressure ($p_{sa}$) and relative humidity (rH)
(4)	$x_{a}=\frac{\left(R_{a}/R_{wv}\times p_{da}\right)}{\left(p_{bar}-p_{da}\right)}$	kg kg^−1^	Mass-related water content depending on partial pressure of water vapour ($p_{da}$) and barometric pressure ($p_{bar}$)
(5)	$\rho_{a\;}=\;\frac{\left(1+x_{a}\right)\times \;p_{bar}}{\left(R_{a}+R_{wv}\times x_{a\;}\right)\times \;\left(273.15+T_{a}\right)\;}$	kg m^−3^	Density of air depending on water content ($x_{a}$), temperature ($T_{a}$) and barometric pressure ($p_{bar}$)
(6)	$x_{a}^{*}=x_{a}\times \rho_{a}$	kg m^−3^	Volume-related water content depending on mass related water content ($x_{a}$) and density ($\rho_{a}$)

*) Calculated approximation equation based on data from Baehr [12].

**) Symbols used in formulas:$\;R_{a}$ is the gas constant of air; $R_{wv}$ is the gas constant of water vapour.

The transpiration rate E in mg water per cm² h can also be determined by simple differential weighing per unit of surface area and time.

(7) $$E=\frac{\Delta FM}{A\times \Delta t}$$

The changes in fresh matter ΔFM were measured by means of a precision balance (0.1 mg). The surface area of the produce item A is determined from produce-specific, easily measurable parameters such as fresh weight (mass, m) and/or possibly geometric dimensions (diameter d, length L) in the preliminary test, as presented in Table 2.

**Table 2:** Correlation between food product mass, diameter and length for surface area determination.

Product	Mass range, m [g]	Surface area, A [cm^2^]	*R*^2^	n
Apple	73–300	A=1.0484449m+61.8323117	0.9913	20
White asparagus	40–85	A=1.427480m+2.917185L−6.453975	0.9888	18
Bell pepper	60–140	A=1.9013m+57.441	0.8400	20
Carrot	80–160	$A=0.4304613m+5.2067765L+0.490815d_{1}-4.13161$	0.9107	20
Radish tuber	6–12	$A=0.57644d_{2}+0.65288d_{3}-14.066$	0.9738	18
Plum	34–96	A=0.63359816m+26.5435856	0.9529	20
Strawberry	7–25	A=1.862363817m+10.6498025	0.9237	20

*n*, number of samples; L, length [cm]; $d_{1}$, mean diameter of minimum 3 measurements over the carrot length [mm]; $d_{2}$, height diameter [mm]; $d_{3}$, transverse diameter [mm], measured twice with a 90° rotation between measurements.

From the measured mean air temperature and relative air humidity at a sufficient distance to the produce surface, the volume-related water content of air (sometimes referred to as absolute humidity, moisture ratio or specific humidity [13]) is determined by applying the known laws of psychrometry. Similarly, the parameters of the air in the intercellular spaces were calculated from the produce temperature at the saturation state. Thus, the total resistance of an individual fruit can be determined by equating and rearranging Eqs. (1) and (7).

The tissue resistance $r_{T}$ of an individual produce item is determined from the difference between the total resistance $r_{tot}$ and the boundary layer resistance $r_{B}$ at unrestricted free convection. The boundary layer resistance characterizes the flow conditions adjacent to the produce surface. This resistance is exclusively dependent on the shape, the dimensions, the surface structure of the object (produce), and the known properties of the fluid (air) [14]. If the boundary layer resistance $r_{B}$ is known, the water vapour concentration at the produce surface $x_{pS}$ can be determined by modification and rearranging of Eq. (1).

(8) $$E=\frac{x_{pS}-x_{a}}{r_{B}}$$

After substituting $x_{pS}$ into Eq. (4) and rearranging, the partial pressure of water vapour is calculated, and afterward, the relative humidity at the produce surface is calculated using Eq. (8).

The following assumptions are made for the calculations:

• The boundary layer resistance ($r_{B}$) of the individual apples with unrestricted free convection is 4.0 s cm^−1^, as long as no available measurements provide differentiated results, e.g., depending on the diameter. The expected error is negligible because tissue resistances ($r_{T}$) greater than 150 s cm^−1^ are expected. From the above-described procedure, the total resistance ($r_{tot}=r_{T}+r_{B}$) is obtained.
• The boundary layer resistance of individual fruits can be determined using two different measuring methods: (1) Forced convection and (2) free convection (wetted produce surface).

(1) Forced convection: forced airflow impinging on the apple to remove the boundary layer resistance ($r_{B}$ = 0 s cm^−1^). The airflow velocity should be greater than 2 m s^−1^.
(2) Free convection: the same goal can be achieved ($r_{B}$ = 0 s cm^−1^) if the fruit to be measured can be wetted with water on its surface. With apples, this is possible by adding a minimal amount of surfactants (e.g., dishwashing liquid). The boundary layer resistance of the individual fruit in free convection can then be determined from the difference between the measurements with authentic $r_{B}$ and with $r_{B}$ = 0.

• The relative air humidity in the intercellular space of the apples is 98.5 % (water activity $a_{w}$ = 0.985) as long as no measured values are available [15]. The expected error in this assumption is negligible as long as the apple shows no external signs of freshness loss (wilting, shrinkage, softening, etc.).
• All resistances are temperature-dependent, requiring measurement at the temperature of interest. Arrhenius's relationship for approximate determination can be used.

## Experimental procedure

The experimental assessment involves the selection and preparation of fruit, the measuring process, and the calculation of tissue resistance and bulk boundary layer, as delineated below.

Preparation of Fruit:

• Identify a group of a minimum of 20 undamaged fruit and systematically label each sample. It is recommended to use fruit from the same harvesting field to avoid introducing variability stemming from diverse agricultural practices, environmental conditions, or genetic variations that may impact the experimental results.
• Expose the chosen fruit to an ambient indoor climate of 20 °C for a minimum of 12 h. Utilize air-permeable materials, such as breathable fabric or mesh netting, to envelop the fruit, effectively minimizing the risk of water loss.

Measuring Process for Individual Fruit:

• Measure the current weight of each identified fruit sample, duly recording the measurement time.
• Place the selected fruit on a wire rack to allow unrestricted free convection in the whole fruit surface during the experiment. Note: no forced convection is employed.
• Record pertinent climate data, such as air temperature and relative humidity, continuously to ensure stable conditions during the experiment. Real-time data or data loggers can be applied to this end.
• Allow the fruit to undergo transpiration for a designated duration, such as 2 h, ensuring unrestricted free convection.
• It is advisable to use infrared sensors to minimize unnecessary handling and ensure accurate measurements.
• Conduct a second measurement of the current weight of each fruit sample and compute the weight loss over the specified time interval.
• Repeat the measurement for at least 24 h.
• Check the average values of air temperature and relative humidity from the recorded data.

Calculation of Tissue Resistance of Individual Fruit:

• Follow the calculation procedure as described in methodological fundamentals.
• Calculate volume-related and mass-related transpiration coefficients ($x_{a}^{*}$, $x_{a}$) using obtained data.

Calculation of Boundary Layer Resistance of Fruit in Bulk:

• Place all fruit together in a package or a box.
• Repeat measurement process for fruit in a package or a box following the process for individual fruit (e.g. measurement of mass variation, air temperature, and relative humidity).
• Extend the period between weight loss measurements for packaged fruit to 2 to 3 days.
• Determine boundary layer resistance ($r_{B}$) from the difference between total resistance in packaging and tissue resistance ($r_{T}$).

## Method validation

**Fig. 2:**
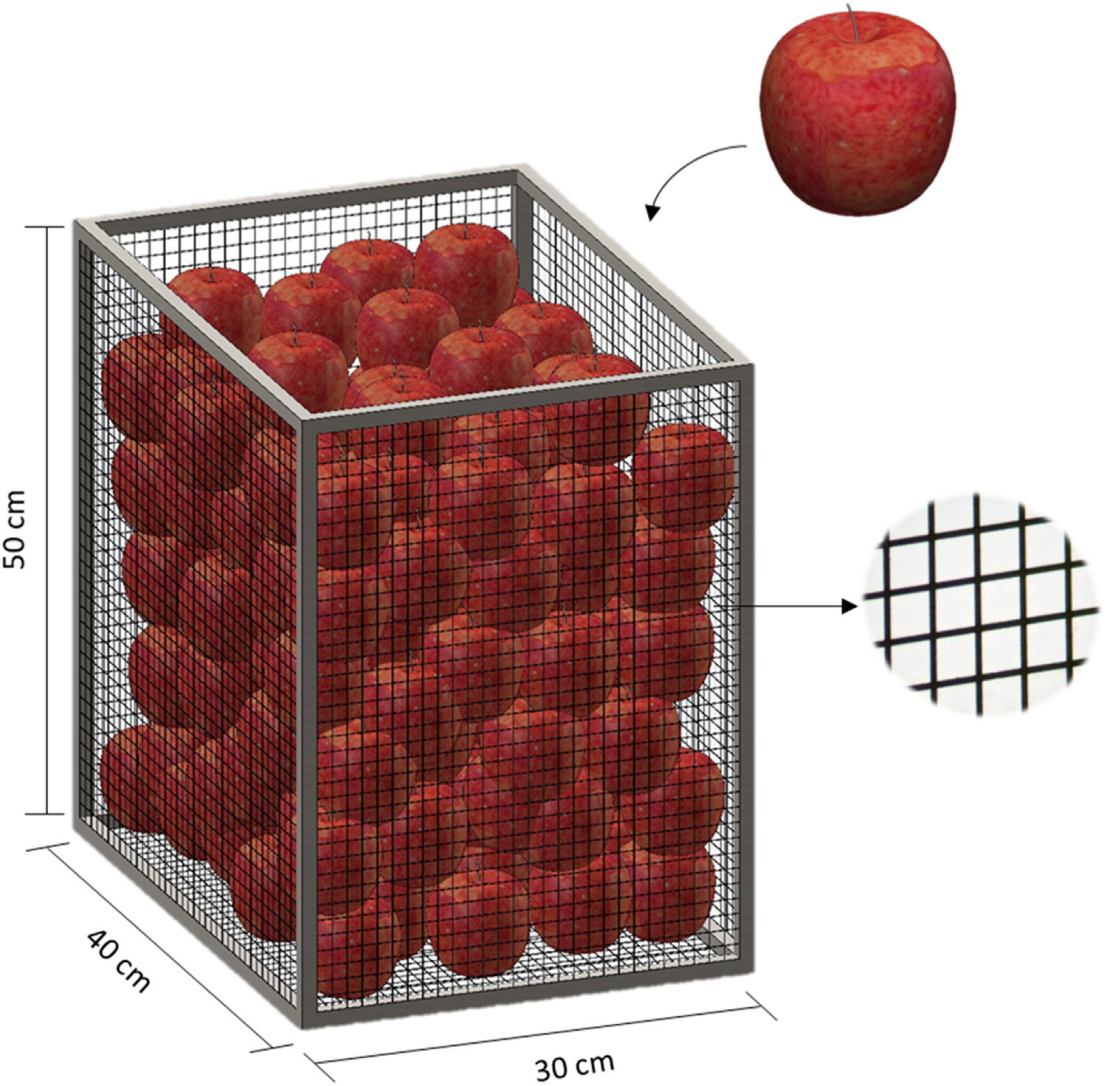
Schematic illustration of the bulk of apples test.

Tissue resistance and boundary layer of apple (cultivar ‘Jonagold’) were determined as individual (20 apples with mass of 131 ± 20 g) and bulk of fruit (circa 20 kg of apples). Tests were done at two different temperatures (20 °C and 1 °C). Experiments at room temperature (20 °C) were done following the experimental procedure while, to perform the tests under cooling, apples were acclimatized (cold room from Frigotec GmbH, Landsberg, Germany) at 1 °C during the preparation of fruit and a test interval of 3 days was considered to detect significant changes in fruit mass. Tests were performed under free (no airflow) and forced convection (airflow of 2 m s^−1^, blower D340/E1 model, Fischbach Luft- und Ventilatorentechnik GmbH, Neunkirchen, Germany), where the total resistance and tissue resistance of apples are obtained, respectively, and the boundary layer can be deduced ($r_{tot}=r_{T}+r_{B}$). The bulk of apples test was conducted inside a box (30 cm × 40 cm × 50 cm) prepared using wire mesh to ensure the free exchange of air between the apples, as shown in Fig. 2. A total of 20 apples were selected randomly within the bulk, representing centralized and corner positions, for tissue resistance and boundary layer determination.

A comprehensive list of different fruit and vegetables and their respective tissue and boundary layer resistances is presented in Table 3. Results show the impact of storage temperature on tissue resistance. The higher the tissue resistance value, the more it will hold water, keeping the fruit and vegetables fresh for longer and avoiding wilting. Apples presented circa 2 times less tissue resistance under 20 °C when compared with apples at 1 °C. By increasing the temperature, the reaction rates can double or even more, reflecting the exponential increase in reaction kinetics as described by the Arrhenius model. This decrease in tissue resistance at warmer temperatures can be is primarily due to increased cell wall flexibility and a higher rate of enzymatic activity, which collectively soften the tissue structure. These results emphasize the importance of determining tissue and boundary layer resistances under specific experimental conditions, as significant variations are likely to occur at different temperatures. Important to highlight that the tissue resistance is also time dependent. During the postharvest phase, tissue resistance increases, in an almost linear relationship [16], due to fruit surface shrinkage. On the other side, as long as the flow conditions do not change, the boundary layer resistance remains constant.

**Table 3:** Comprehensive list of different fruit and vegetables and their respective tissue and boundary layer resistances.

Food produce	Tissue resistance [s cm^−1^]*	Boundary Layer Resistance [s cm^−1^]’	Source
Apple (cultivar ‘Jonagold’) at 20 °C	391 ± 67	4.8	Measurement
Apple (cultivar ‘Jonagold’) at 1 °C	750 ± 96	5.2	Measurement
Strawberry	3–23	–	Bovi et al. [18]
Plum	23–38	–	
Radish tuber	0.25–1.5	1.0–1.5	Linke and Geyer [16]
Carrot	1–6	1.2–2.4	
Apple	170–320	3.0–4.0	
White asparagus	11–12.5	1.0–2.0	
Bell pepper	35–80	3.0–4.5	
Bulk of apples (cultivar ‘Jonagold’) at 20°C	363 ± 45	50.4	Measurement
Bunch radishes in perforated film	–	18.8	Linke and Geyer [16]
Bunch radishes in nearly impermeable film	–	820	

⁎ All values reported from the literature [3,16] were performed with freshly harvested fruit and vegetables, while the tests performed for this study used apples stored in unknown conditions (commercial fruit). For this reason, higher values were obtained.

The non-destructive method developed at ATB is a highly effective tool for the configuration of technical facilities (such as cooling equipment and packaging materials) and technological procedures (including washing, precooling, and storage) in postharvest preservation. A key advantage of this method is its ability to account for the initial properties of the produce as well as the changes that occur during postharvest, using the produce itself as a sensor for maintaining quality.

## Limitations

None.

## Ethics statements

Not applicable.

## CRediT authorship contribution statement

**Manfred Linke:** Conceptualization, Methodology, Writing – original draft. **Tuany Gabriela Hoffmann:** Investigation, Writing – review & editing. **Akshay D. Sonawane:** Investigation, Writing – review & editing. **Guido Rux:** Investigation, Validation. **Pramod V. Mahajan:** Funding acquisition, Methodology, Writing – original draft, Writing – review & editing.

## Declaration of competing interest

The authors declare that they have no known competing financial interests or personal relationships that could have appeared to influence the work reported in this paper.

## Data Availability

Data will be made available on request.

## Appendix Supplementary materials

Image, application 1
